# Supplementary material for: Large-scale language analysis of peer review reports
Source: eLife. 2020 Jul 17;9:e53249. doi: 10.7554/eLife.53249 (PMC7390598; doi:10.7554/eLife.53249)
Supplement: Supplementary file 1. [file elife-53249-supp1.docx]

**Buljan et al. 2020 eLife 9:e53249. Supplementary file 1**

**Word count (figure 1): summary data and mixed model linear regression coefficients and residuals**

**Table 1.** LIWC **Word count** in review reports per reviewer recommendation, journal’s field of research, type of peer review type and reviewer’s gender

| **Reviewer recommendation** | **Journal’s field of research** | **Peer review type** | **Reviewer gender** | **N** | **Predicted mean** | **Lower 95% CI** | **Upper 95% CI** |
| --- | --- | --- | --- | --- | --- | --- | --- |
| Accept | HMS | Double-blind | Female | 729 | 134.643 | 57.608 | 211.677 |
|  |  |  | Male | 3044 | 108.630 | 31.608 | 185.653 |
|  |  | Single-blind | Female | 1526 | 130.690 | 69.525 | 191.855 |
|  |  |  | Male | 5113 | 104.677 | 43.531 | 165.824 |
|  | LS | Double-blind | Female | 89 | 232.450 | 110.788 | 354.111 |
|  |  |  | Male | 255 | 206.437 | 84.782 | 328.093 |
|  |  | Single-blind | Female | 201 | 228.497 | 122.692 | 334.301 |
|  |  |  | Male | 478 | 202.484 | 96.688 | 308.280 |
|  | PS | Double-blind | Female | 16 | 178.666 | 90.528 | 266.804 |
|  |  |  | Male | 92 | 152.653 | 64.529 | 240.778 |
|  |  | Single-blind | Female | 2669 | 174.713 | 124.277 | 225.149 |
|  |  |  | Male | 11591 | 148.701 | 98.292 | 199.110 |
|  | SS&E | Double-blind | Female | 221 | 310.634 | 188.236 | 433.031 |
|  |  |  | Male | 193 | 284.621 | 162.230 | 407.012 |
|  |  | Single-blind | Female | 20 | 306.681 | 184.254 | 429.108 |
|  |  |  | Male | 150 | 280.668 | 158.249 | 403.088 |
| Minor revision | HMS | Double-blind | Female | 737 | 314.329 | 237.353 | 391.304 |
|  |  |  | Male | 2151 | 288.316 | 211.352 | 365.281 |
|  |  | Single-blind | Female | 7983 | 310.376 | 249.293 | 371.459 |
|  |  |  | Male | 23822 | 284.364 | 223.298 | 345.429 |
|  | LS | Double-blind | Female | 827 | 412.136 | 290.514 | 533.757 |
|  |  |  | Male | 1532 | 386.123 | 264.507 | 507.739 |
|  |  | Single-blind | Female | 1924 | 408.183 | 302.429 | 513.937 |
|  |  |  | Male | 3925 | 382.170 | 276.424 | 487.917 |
|  | PS | Double-blind | Female | 102 | 358.352 | 270.265 | 446.439 |
|  |  |  | Male | 251 | 332.340 | 244.266 | 420.413 |
|  |  | Single-blind | Female | 24506 | 354.399 | 304.063 | 404.735 |
|  |  |  | Male | 84040 | 328.387 | 278.076 | 378.697 |
|  | SS&E | Double-blind | Female | 3939 | 490.320 | 367.962 | 612.677 |
|  |  |  | Male | 3902 | 464.307 | 341.956 | 586.659 |
|  |  | Single-blind | Female | 447 | 486.367 | 363.984 | 608.750 |
|  |  |  | Male | 1608 | 460.355 | 337.979 | 582.730 |
| Major revision | HMS | Double-blind | Female | 3242 | 476.087 | 399.110 | 553.064 |
|  |  |  | Male | 7756 | 450.075 | 373.108 | 527.041 |
|  |  | Single-blind | Female | 10327 | 472.135 | 411.052 | 533.217 |
|  |  |  | Male | 26235 | 446.122 | 385.055 | 507.189 |
|  | LS | Double-blind | Female | 579 | 573.894 | 452.274 | 695.514 |
|  |  |  | Male | 1175 | 547.882 | 426.267 | 669.496 |
|  |  | Single-blind | Female | 1379 | 569.941 | 464.191 | 675.692 |
|  |  |  | Male | 2855 | 543.929 | 438.185 | 649.673 |
|  | PS | Double-blind | Female | 60 | 520.111 | 432.026 | 608.195 |
|  |  |  | Male | 196 | 494.098 | 406.025 | 582.171 |
|  |  | Single-blind | Female | 16225 | 516.158 | 465.827 | 566.489 |
|  |  |  | Male | 59842 | 490.145 | 439.839 | 540.452 |
|  | SS&E | Double-blind | Female | 2017 | 652.078 | 529.724 | 774.432 |
|  |  |  | Male | 1852 | 626.066 | 503.717 | 748.414 |
|  |  | Single-blind | Female | 212 | 648.126 | 525.747 | 770.504 |
|  |  |  | Male | 906 | 622.113 | 499.741 | 744.485 |
| Reject | HMS | Double-blind | Female | 3752 | 389.055 | 312.079 | 466.032 |
|  |  |  | Male | 14118 | 363.043 | 286.079 | 440.007 |
|  |  | Single-blind | Female | 7592 | 385.103 | 324.018 | 446.187 |
|  |  |  | Male | 27961 | 359.090 | 298.024 | 420.157 |
|  | LS | Double-blind | Female | 475 | 486.862 | 365.241 | 608.483 |
|  |  |  | Male | 1028 | 460.850 | 339.235 | 582.465 |
|  |  | Single-blind | Female | 1312 | 482.910 | 377.156 | 588.664 |
|  |  |  | Male | 3110 | 456.897 | 351.151 | 562.643 |
|  | PS | Double-blind | Female | 80 | 433.079 | 344.992 | 521.165 |
|  |  |  | Male | 233 | 407.066 | 318.993 | 495.139 |
|  |  | Single-blind | Female | 16139 | 429.126 | 378.788 | 479.464 |
|  |  |  | Male | 64573 | 403.114 | 352.803 | 453.424 |
|  | SS&E | Double-blind | Female | 2628 | 565.046 | 442.694 | 687.399 |
|  |  |  | Male | 3451 | 539.034 | 416.688 | 661.380 |
|  |  | Single-blind | Female | 638 | 561.094 | 438.715 | 683.473 |
|  |  |  | Male | 2418 | 535.081 | 412.710 | 657.452 |

LIWC – Linguistic Inquiry and Word Count software, HMS – Health and Medical Sciences, LS – Life Sciences, PS – Physical sciences, SS&E – Social Sciences and Economics

**Table 2.** LIWC **Word Count** mixed model linear regression coefficients and residuals

| Fixed effects | | Standardized estimate | 95% CI | | P |
| --- | --- | --- | --- | --- | --- |
|  | |  | Lower | Upper |  |
|  | (Intercept) | 134.6 | 57.6 | 211.7 | <0.001 |
| Journal’s field of research (reference HMS) | |  |  |  |  |
|  | Life sciences | 97.8 | -14.0 | 209.7 | 0.090 |
|  | Physical sciences | 44.0 | -22.1 | 110.1 | 0.200 |
|  | Social sciences and economics | 176.0 | 52.9 | 299.1 | 0.010 |
| Reviewer recommendation (Reference Accept) | |  |  |  |  |
|  | Minor revision | 179.7 | 175.8 | 183.6 | <0.001 |
|  | Major revision | 341.4 | 337.6 | 345.3 | <0.001 |
|  | Reject | 254.4 | 250.6 | 258.3 | <0.001 |
| Gender: Male | | -26.0 | -28.0 | -24.0 | <0.001 |
| Type of Peer review: Single blind | | -4.0 | -81.5 | 73.6 | 0.920 |
|  | |  |  |  |  |
|  | |  |  |  |  |
| Random effects | | Standard deviation |  |  |  |
| Journal | | 114.7 |  |  |  |
| Article Type | | 20.62 |  |  |  |
| Residual | | 293.2 |  |  |  |

LIWC – Linguistic Inquiry and Word Count software, CI – confidence interval, HMS – Health and Medical Sciences
