# Supplementary material for: Large-scale language analysis of peer review reports
Source: eLife. 2020 Jul 17;9:e53249. doi: 10.7554/eLife.53249 (PMC7390598; doi:10.7554/eLife.53249)
Supplement: Supplementary file 2. [file elife-53249-supp2.docx]

**Buljan et al. 2020 eLife 9:e53249. Supplementary file 2**

**Analytical tone (figure 2A): summary data, mixed model linear regression coefficients and residuals, and examples of reports with high and low scores for LIWC analytical tone**

**Table 3.** Average LIWC **Analytical** tone levels in review reports per reviewer recommendation, journal’s field of research, type of peer review type and reviewer’s gender (range 0-100)

| **Reviewer recommendation** | **Journal’s field of research** | **Peer review type** | **Reviewer gender** | **N** | **Predicted mean** | **Lower 95% CI** | **Upper 95% CI** |
| --- | --- | --- | --- | --- | --- | --- | --- |
| Accept | HMS | Double-blind | Female | 729 | 87.003 | 85.742 | 88.263 |
|  |  |  | Male | 3044 | 86.888 | 85.629 | 88.148 |
|  |  | Single-blind | Female | 1526 | 88.846 | 87.882 | 89.809 |
|  |  |  | Male | 5113 | 88.731 | 87.769 | 89.693 |
|  | LS | Double-blind | Female | 89 | 88.758 | 86.708 | 90.808 |
|  |  |  | Male | 255 | 88.644 | 86.594 | 90.693 |
|  |  | Single-blind | Female | 201 | 90.601 | 88.832 | 92.370 |
|  |  |  | Male | 478 | 90.487 | 88.719 | 92.255 |
|  | PS | Double-blind | Female | 16 | 87.814 | 86.351 | 89.277 |
|  |  |  | Male | 92 | 87.700 | 86.238 | 89.162 |
|  |  | Single-blind | Female | 2669 | 89.657 | 88.901 | 90.413 |
|  |  |  | Male | 11591 | 89.543 | 88.789 | 90.296 |
|  | SS&E | Double-blind | Female | 221 | 88.120 | 86.060 | 90.180 |
|  |  |  | Male | 193 | 88.006 | 85.946 | 90.065 |
|  |  | Single-blind | Female | 20 | 89.963 | 87.900 | 92.025 |
|  |  |  | Male | 150 | 89.848 | 87.787 | 91.910 |
| Minor revision | HMS | Double-blind | Female | 737 | 88.062 | 86.807 | 89.316 |
|  |  |  | Male | 2151 | 87.947 | 86.694 | 89.201 |
|  |  | Single-blind | Female | 7983 | 89.905 | 88.949 | 90.860 |
|  |  |  | Male | 23822 | 89.790 | 88.836 | 90.744 |
|  | LS | Double-blind | Female | 827 | 89.817 | 87.771 | 91.863 |
|  |  |  | Male | 1532 | 89.703 | 87.657 | 91.749 |
|  |  | Single-blind | Female | 1924 | 91.660 | 89.896 | 93.424 |
|  |  |  | Male | 3925 | 91.546 | 89.782 | 93.309 |
|  | PS | Double-blind | Female | 102 | 88.873 | 87.415 | 90.331 |
|  |  |  | Male | 251 | 88.759 | 87.302 | 90.216 |
|  |  | Single-blind | Female | 24506 | 90.716 | 89.971 | 91.461 |
|  |  |  | Male | 84040 | 90.601 | 89.859 | 91.344 |
|  | SS&E | Double-blind | Female | 3939 | 89.179 | 87.123 | 91.235 |
|  |  |  | Male | 3902 | 89.064 | 87.009 | 91.120 |
|  |  | Single-blind | Female | 447 | 91.022 | 88.963 | 93.080 |
|  |  |  | Male | 1608 | 90.907 | 88.850 | 92.965 |
| Major revision | HMS | Double-blind | Female | 3242 | 87.402 | 86.148 | 88.656 |
|  |  |  | Male | 7756 | 87.287 | 86.034 | 88.541 |
|  |  | Single-blind | Female | 10327 | 89.245 | 88.290 | 90.199 |
|  |  |  | Male | 26235 | 89.130 | 88.177 | 90.084 |
|  | LS | Double-blind | Female | 579 | 89.157 | 87.112 | 91.203 |
|  |  |  | Male | 1175 | 89.043 | 86.998 | 91.088 |
|  |  | Single-blind | Female | 1379 | 91.000 | 89.237 | 92.764 |
|  |  |  | Male | 2855 | 90.886 | 89.123 | 92.649 |
|  | PS | Double-blind | Female | 60 | 88.213 | 86.756 | 89.671 |
|  |  |  | Male | 196 | 88.099 | 86.642 | 89.555 |
|  |  | Single-blind | Female | 16225 | 90.056 | 89.312 | 90.800 |
|  |  |  | Male | 59842 | 89.942 | 89.200 | 90.683 |
|  | SS&E | Double-blind | Female | 2017 | 88.519 | 86.463 | 90.575 |
|  |  |  | Male | 1852 | 88.405 | 86.349 | 90.460 |
|  |  | Single-blind | Female | 212 | 90.362 | 88.304 | 92.420 |
|  |  |  | Male | 906 | 90.247 | 88.190 | 92.305 |
| Reject | HMS | Double-blind | Female | 3752 | 86.762 | 85.508 | 88.016 |
|  |  |  | Male | 14118 | 86.648 | 85.395 | 87.901 |
|  |  | Single-blind | Female | 7592 | 88.605 | 87.650 | 89.560 |
|  |  |  | Male | 27961 | 88.491 | 87.537 | 89.444 |
|  | LS | Double-blind | Female | 475 | 88.518 | 86.472 | 90.564 |
|  |  |  | Male | 1028 | 88.403 | 86.358 | 90.449 |
|  |  | Single-blind | Female | 1312 | 90.361 | 88.597 | 92.124 |
|  |  |  | Male | 3110 | 90.246 | 88.483 | 92.009 |
|  | PS | Double-blind | Female | 80 | 87.574 | 86.116 | 89.031 |
|  |  |  | Male | 233 | 87.459 | 86.002 | 88.916 |
|  |  | Single-blind | Female | 16139 | 89.416 | 88.672 | 90.161 |
|  |  |  | Male | 64573 | 89.302 | 88.560 | 90.044 |
|  | SS&E | Double-blind | Female | 2628 | 87.879 | 85.824 | 89.935 |
|  |  |  | Male | 3451 | 87.765 | 85.710 | 89.820 |
|  |  | Single-blind | Female | 638 | 89.722 | 87.664 | 91.780 |
|  |  |  | Male | 2418 | 89.608 | 87.551 | 91.665 |

LIWC – Linguistic Inquiry and Word Count software, HMS – Health and Medical Sciences, LS – Life Sciences, PS – Physical sciences, SS&E – Social Sciences and Economics

**Table 4.** LIWC **Analytic** tone mixed model linear regression coefficients and residuals

| Fixed effects | | Standardized estimate | 95% CI | | P |
| --- | --- | --- | --- | --- | --- |
|  | |  | Lower | Upper |  |
|  | (Intercept) | 87.0 | 85.7 | 88.3 | <0.001 |
| Journal’s field of research (reference HMS) | |  |  |  |  |
|  | Life sciences | 1.76 | -0.2 | 3.7 | 0.080 |
|  | Physical sciences | 0.81 | -0.3 | 1.9 | 0.170 |
|  | Social sciences and economics | 1.12 | -1.0 | 3.2 | 0.310 |
| Reviewer recommendation (Reference Accept) | |  |  |  |  |
|  | Minor revision | 1.1 | 0.9 | 1.2 | <0.001 |
|  | Major revision | 0.4 | 0.3 | 0.6 | <0.001 |
|  | Reject | -0.2 | -0.4 | -0.1 | <0.001 |
| Gender: Male | | -0.1 | -0.2 | -0.1 | <0.001 |
| Peer review type: Single blind | | 1.8 | 0.5 | 3.2 | 0.010 |
|  | |  |  |  |  |
|  | |  |  |  |  |
| Random effects | | Standard deviation |  |  |  |
| LIWC Word count | | 2.44 |  |  |  |
| Journal | | 1.96 |  |  |  |
| Article type | | 0.12 |  |  |  |
| Residual | | 10.6 |  |  |  |

LIWC – Linguistic Inquiry and Word Count software, CI – confidence interval, HMS – Health and Medical Sciences

**Table 5.** Examples of review reports with high and low scores for LIWC **Analytic** tone

| **High** |
| --- |
| The authors should comment the [anonymized] efficiency of the proposed technique. The authors should use a statistical test for the comparisons between the techniques in their experiments. A larger [anonymized] should be also used for testing the [anonymized] of the proposed technique. |
| 1. Please comment on the high rate of death in [anonymized]? Was this true of [anonymized] performed at [anonymized]? Please add this data 2. The data seem to indicate that if you want a lower [anonymized] rate the children need to be seen at a hospital with appropriate [anonymized]. Please comment on this as this stands out in the data. |
| The authors present a study on the development of novel alternative technology for [anonymized]. The results are interesting. The manuscript is generally well prepared except for the Results section. A major revision in the Results section is needed, more specifically, a simple description of each of the Figures and Tables are needed, before being considered for publication. |
| This paper addresses an interesting application of the [anonymized]onto [anonymized]. The manuscript presents new results and is suitable for the publication after minor revision. However, the authors asked to respond to the following minor comments:. 1. Check the scheme of Fig. 2 - ' [anonymized]' or ' [anonymized]'?. . |
| The author must complete the following:. 1- Correction for many words and sentences that mentioned in the attachment. 2- The results must showed as curves in addition to tables. 3- Repeat the revised copy with addition of scientific equations. 4- The references are not updated. |
|  |
| **Low** |
| Your paper is well-written and I enjoyed reading it. The one suggestion I have is that it would be good if you could clearly identify which of your results are new and which ones are replications of prior results. |
| I see that the study has been done quiet methodically. However I am not convinced with the logic behind this study? Why was this study done, what problem does it really address? |
| This is a very simple, yet well planned and written study. Though it is not novel in concept, there remains enough controversy in this area that I believe your paper will be helpful. |
| The paper is so poorly written. Introduction only has one paragraph and the meaning and novelty of this study is not mentioned. Results are not enough and discussions are weak. Thus I think it is not acceptable. |
| Authors did not show any results. They just developed their model but, how do I know that it is correct? I need comparisons with actual data or with another model. They need to work better this point. |
