# Supplementary material for: Large-scale language analysis of peer review reports
Source: eLife. 2020 Jul 17;9:e53249. doi: 10.7554/eLife.53249 (PMC7390598; doi:10.7554/eLife.53249)
Supplement: Supplementary file 3. [file elife-53249-supp3.docx]

**Buljan et al. 2020 eLife 9:e53249. Supplementary file 3**

**Clout (figure 2B): summary data, mixed model linear regression coefficients and residuals, and examples of reports with high and low scores for LIWC clout**

**Table 6.** Average LIWC **Clout** tone levels in review reports per reviewer recommendation, journal’s field of research, type of peer review type and reviewer’s gender (range 0-100)

| **Reviewer recommendation** | **Journal’s field of research** | **Peer review type** | **Reviewer gender** | **N** | **Predicted mean** | **Lower 95% CI** | **Upper 95% CI** |
| --- | --- | --- | --- | --- | --- | --- | --- |
| Accept | HMS | Double-blind | Female | 729 | 51.222 | 49.921 | 52.522 |
|  |  |  | Male | 3044 | 50.174 | 48.874 | 51.473 |
|  |  | Single-blind | Female | 1526 | 49.840 | 48.855 | 50.825 |
|  |  |  | Male | 5113 | 48.792 | 47.809 | 49.775 |
|  | LS | Double-blind | Female | 89 | 49.853 | 47.724 | 51.981 |
|  |  |  | Male | 255 | 48.805 | 46.676 | 50.933 |
|  |  | Single-blind | Female | 201 | 48.471 | 46.637 | 50.304 |
|  |  |  | Male | 478 | 47.423 | 45.590 | 49.256 |
|  | PS | Double-blind | Female | 16 | 49.000 | 47.485 | 50.514 |
|  |  |  | Male | 92 | 47.952 | 46.439 | 49.465 |
|  |  | Single-blind | Female | 2669 | 47.618 | 46.856 | 48.380 |
|  |  |  | Male | 11591 | 46.570 | 45.811 | 47.330 |
|  | SS&E | Double-blind | Female | 221 | 54.319 | 52.180 | 56.458 |
|  |  |  | Male | 193 | 53.271 | 51.133 | 55.409 |
|  |  | Single-blind | Female | 20 | 52.937 | 50.796 | 55.079 |
|  |  |  | Male | 150 | 51.889 | 49.748 | 54.030 |
| Minor revision | HMS | Double-blind | Female | 737 | 49.968 | 48.675 | 51.262 |
|  |  |  | Male | 2151 | 48.921 | 47.628 | 50.213 |
|  |  | Single-blind | Female | 7983 | 48.587 | 47.612 | 49.562 |
|  |  |  | Male | 23822 | 47.539 | 46.565 | 48.512 |
|  | LS | Double-blind | Female | 827 | 48.599 | 46.475 | 50.723 |
|  |  |  | Male | 1532 | 47.551 | 45.427 | 49.675 |
|  |  | Single-blind | Female | 1924 | 47.217 | 45.389 | 49.045 |
|  |  |  | Male | 3925 | 46.170 | 44.342 | 47.997 |
|  | PS | Double-blind | Female | 102 | 47.747 | 46.238 | 49.255 |
|  |  |  | Male | 251 | 46.699 | 45.191 | 48.206 |
|  |  | Single-blind | Female | 24506 | 46.365 | 45.616 | 47.114 |
|  |  |  | Male | 84040 | 45.317 | 44.571 | 46.063 |
|  | SS&E | Double-blind | Female | 3939 | 53.066 | 50.932 | 55.200 |
|  |  |  | Male | 3902 | 52.018 | 49.884 | 54.151 |
|  |  | Single-blind | Female | 447 | 51.684 | 49.547 | 53.821 |
|  |  |  | Male | 1608 | 50.636 | 48.500 | 52.772 |
| Major revision | HMS | Double-blind | Female | 3242 | 47.483 | 46.190 | 48.776 |
|  |  |  | Male | 7756 | 46.435 | 45.143 | 47.727 |
|  |  | Single-blind | Female | 10327 | 46.101 | 45.127 | 47.076 |
|  |  |  | Male | 26235 | 45.053 | 44.080 | 46.026 |
|  | LS | Double-blind | Female | 579 | 46.114 | 43.990 | 48.238 |
|  |  |  | Male | 1175 | 45.066 | 42.942 | 47.189 |
|  |  | Single-blind | Female | 1379 | 44.732 | 42.904 | 46.560 |
|  |  |  | Male | 2855 | 43.684 | 41.857 | 45.511 |
|  | PS | Double-blind | Female | 60 | 45.261 | 43.753 | 46.769 |
|  |  |  | Male | 196 | 44.213 | 42.706 | 45.720 |
|  |  | Single-blind | Female | 16225 | 43.879 | 43.131 | 44.627 |
|  |  |  | Male | 59842 | 42.831 | 42.086 | 43.577 |
|  | SS&E | Double-blind | Female | 2017 | 50.580 | 48.447 | 52.714 |
|  |  |  | Male | 1852 | 49.532 | 47.399 | 51.665 |
|  |  | Single-blind | Female | 212 | 49.198 | 47.062 | 51.335 |
|  |  |  | Male | 906 | 48.150 | 46.015 | 50.286 |
| Reject | HMS | Double-blind | Female | 3752 | 42.789 | 41.496 | 44.082 |
|  |  |  | Male | 14118 | 41.741 | 40.449 | 43.033 |
|  |  | Single-blind | Female | 7592 | 41.407 | 40.433 | 42.382 |
|  |  |  | Male | 27961 | 40.359 | 39.386 | 41.332 |
|  | LS | Double-blind | Female | 475 | 41.420 | 39.296 | 43.544 |
|  |  |  | Male | 1028 | 40.372 | 38.248 | 42.496 |
|  |  | Single-blind | Female | 1312 | 40.038 | 38.210 | 41.866 |
|  |  |  | Male | 3110 | 38.990 | 37.163 | 40.817 |
|  | PS | Double-blind | Female | 80 | 40.567 | 39.059 | 42.075 |
|  |  |  | Male | 233 | 39.519 | 38.012 | 41.026 |
|  |  | Single-blind | Female | 16139 | 39.185 | 38.437 | 39.934 |
|  |  |  | Male | 64573 | 38.138 | 37.392 | 38.883 |
|  | SS&E | Double-blind | Female | 2628 | 45.886 | 43.753 | 48.020 |
|  |  |  | Male | 3451 | 44.838 | 42.706 | 46.971 |
|  |  | Single-blind | Female | 638 | 44.505 | 42.368 | 46.641 |
|  |  |  | Male | 2418 | 43.457 | 41.321 | 45.592 |

LIWC – Linguistic Inquiry and Word Count software, HMS – Health and Medical Sciences, LS – Life Sciences, PS – Physical sciences, SS&E – Social Sciences and Economics

**Table 7.** LIWC **Clout** tone mixed model linear regression coefficients and residuals

| Fixed effects | | Standardized estimate | 95% CI | | P |
| --- | --- | --- | --- | --- | --- |
|  | |  | Lower | Upper |  |
|  | (Intercept) | 51.2 | 49.9 | 52.5 | 0.001 |
| Journal’s field of research (reference HMS) | |  |  |  |  |
|  | Life sciences | -1.4 | -3.4 | 0.6 | 0.190 |
|  | Physical sciences | -2.2 | -3.4 | -1.0 | 0.001 |
|  | Social sciences and economics | 3.1 | 0.9 | 5.3 | 0.010 |
| Reviewer recommendation (Reference Accept) | |  |  |  |  |
|  | Minor revision | -1.3 | -1.4 | -1.1 | 0.001 |
|  | Major revision | -3.7 | -3.9 | -3.6 | 0.001 |
|  | Reject | -8.4 | -8.6 | -8.3 | 0.001 |
| Gender: Male | | -1.1 | -1.1 | -1.0 | 0.001 |
| Peer review type: Single blind | | -1.4 | -2.8 | 0.1 | 0.060 |
|  | |  |  |  |  |
|  | |  |  |  |  |
| Random effects | | Standard deviation |  |  |  |
| LIWC Word count | | 1.2 |  |  |  |
| Journal | | 2.0 |  |  |  |
| Article type | | 0.0 |  |  |  |
| Residual | | 12.0 |  |  |  |

LIWC – Linguistic Inquiry and Word Count software, CI – confidence interval, HMS – Health and Medical Sciences

**Table 8.** Examples of review reports with high and low scores for LIWC **Clout** tone

| **High** |
| --- |
| Abstract introduction too long and unnecessary. Key-words revision and show the index medicus list. The methodology of your abstract could be the purpose of your study. Write the material and methodology you have employed for your paper. No results are presented. One conclusion is necessary. Manuscript: Define the objective of your study. Methodology and results chapters are confusing. Describe how you analyzed your data, how you do your analysis it, and the statistical analysis you have performed. Present clear your results. Discussion: try to compare your results with another studies and publications. |
| Excellent work. Only you should be doing an up-to-date of references, there are more in the world wide literature, discuss them, and that could help to improve the quality of your article. |
| You describe a simple and easily calculated score for prognostication [anonymized]. I think the methodology and discussion are relevant and honest. The high false positive rate is a concern and you address that in your discussion. I would also recommend that you include a statement in your discussion and conclusion that further studies using this score are required before this score can be recommended for general use. |
| The artice is well written and interesting. You should, however, discuss more the [anonymized] methods that you used for testing. Especially [anonymized]is interesting, because it outperformed your method. |
| I applaud your effort to address a highly relevant issue that continues to compromise and/or obstruct societal and legal efforts to protect children from repetitive abusive trauma. Your manuscript is well-written, your methods appear sound, your tables and figures add value, your results are compelling, your conclusions are supported by your results, and the limitations of your study are discussed thoroughly and objectively. Having read your manuscript several times, I can suggest no specific edits that will improve your submission. Thank you for this compelling and important work. |
|  |
| **Low** |
| Readers cannot find the scientific innovation and novelty from this manuscript. Experiment was not carefully designed and experiment data is not enough. Moreover, the manuscript was not carefully prepared. The reference format is not correct. |
| I think the paper is not within the scope of this journal. It makes a confusion between quantitative and qualitative information and data. The conclusion is unsubstantial. Many sentences are not really conclusions. |
| Well-done study despite small sample size. Statistics should be reviewed by a qualified statistician. Do not know why median scores were used rather than means. Tables were small and not easy to read. |
| I have carefully read this paper and I have appreciated both the hot topic and the clear analysis. I have no suggestions, I think that the paper is interesting and well written. I cannot check the English because I am not a mother tongue speaker. |
| Part of the title and summary is not bad, but there is not detailed information in other sections If I wanted to work on this manuscript, I would not get many information (For example; data which are used for optimization) The manuscript unassociated with (or was not compared) other articles. |
