# Supplementary material for: Large-scale language analysis of peer review reports
Source: eLife. 2020 Jul 17;9:e53249. doi: 10.7554/eLife.53249 (PMC7390598; doi:10.7554/eLife.53249)
Supplement: Supplementary file 4. [file elife-53249-supp4.docx]

**Buljan et al. 2020 eLife 9:e53249. Supplementary file 4**

**Authenticity (figure 2C): summary data, mixed model linear regression coefficients and residuals, and examples of reports with high and low scores for LIWC authenticity**

**Table 9.** Average LIWC **Authenticity** tone levels in review reports per reviewer recommendation, journal’s field of research, type of peer review type and reviewer’s gender (range 0-100)

| **Reviewer recommendation** | **Journal’s field of research** | **Peer review type** | **Reviewer gender** | **N** | **Predicted mean** | **Lower 95% CI** | **Upper 95% CI** |
| --- | --- | --- | --- | --- | --- | --- | --- |
| Accept | HMS | Double-blind | female | 729 | 20.660 | 18.462 | 22.859 |
|  |  |  | male | 3044 | 20.744 | 18.547 | 22.942 |
|  |  | Single-blind | female | 1526 | 20.397 | 18.730 | 22.064 |
|  |  |  | male | 5113 | 20.481 | 18.815 | 22.146 |
|  | LS | Double-blind | female | 89 | 19.962 | 16.351 | 23.572 |
|  |  |  | male | 255 | 20.046 | 16.435 | 23.656 |
|  |  | Single-blind | female | 201 | 19.698 | 16.587 | 22.809 |
|  |  |  | male | 478 | 19.782 | 16.672 | 22.893 |
|  | PS | Double-blind | female | 16 | 20.318 | 17.758 | 22.877 |
|  |  |  | male | 92 | 20.402 | 17.843 | 22.960 |
|  |  | Single-blind | female | 2669 | 20.054 | 18.765 | 21.344 |
|  |  |  | male | 11591 | 20.138 | 18.851 | 21.425 |
|  | SS&E | Double-blind | female | 221 | 25.673 | 22.042 | 29.304 |
|  |  |  | male | 193 | 25.757 | 22.127 | 29.387 |
|  |  | Single-blind | female | 20 | 25.410 | 21.776 | 29.043 |
|  |  |  | male | 150 | 25.494 | 21.860 | 29.127 |
| Minor revision | HMS | Double-blind | female | 737 | 19.595 | 17.404 | 21.787 |
|  |  |  | male | 2151 | 19.679 | 17.489 | 21.870 |
|  |  | Single-blind | female | 7983 | 19.332 | 17.675 | 20.989 |
|  |  |  | male | 23822 | 19.416 | 17.760 | 21.071 |
|  | LS | Double-blind | female | 827 | 18.897 | 15.290 | 22.503 |
|  |  |  | male | 1532 | 18.981 | 15.375 | 22.587 |
|  |  | Single-blind | female | 1924 | 18.633 | 15.528 | 21.739 |
|  |  |  | male | 3925 | 18.717 | 15.612 | 21.823 |
|  | PS | Double-blind | female | 102 | 19.253 | 16.699 | 21.807 |
|  |  |  | male | 251 | 19.337 | 16.784 | 21.890 |
|  |  | Single-blind | female | 24506 | 18.990 | 17.713 | 20.266 |
|  |  |  | male | 84040 | 19.074 | 17.799 | 20.348 |
|  | SS&E | Double-blind | female | 3939 | 24.608 | 20.982 | 28.235 |
|  |  |  | male | 3902 | 24.692 | 21.066 | 28.318 |
|  |  | Single-blind | female | 447 | 24.345 | 20.716 | 27.974 |
|  |  |  | male | 1608 | 24.429 | 20.800 | 28.057 |
| Major revision | HMS | Double-blind | female | 3242 | 19.561 | 17.369 | 21.752 |
|  |  |  | male | 7756 | 19.645 | 17.454 | 21.835 |
|  |  | Single-blind | female | 10327 | 19.297 | 17.641 | 20.954 |
|  |  |  | male | 26235 | 19.381 | 17.726 | 21.036 |
|  | LS | Double-blind | female | 579 | 18.862 | 15.256 | 22.468 |
|  |  |  | male | 1175 | 18.946 | 15.341 | 22.552 |
|  |  | Single-blind | female | 1379 | 18.599 | 15.493 | 21.704 |
|  |  |  | male | 2855 | 18.683 | 15.578 | 21.788 |
|  | PS | Double-blind | female | 60 | 19.218 | 16.665 | 21.772 |
|  |  |  | male | 196 | 19.302 | 16.750 | 21.855 |
|  |  | Single-blind | female | 16225 | 18.955 | 17.679 | 20.231 |
|  |  |  | male | 59842 | 19.039 | 17.766 | 20.312 |
|  | SS&E | Double-blind | female | 2017 | 24.574 | 20.948 | 28.200 |
|  |  |  | male | 1852 | 24.658 | 21.032 | 28.283 |
|  |  | Single-blind | female | 212 | 24.310 | 20.682 | 27.939 |
|  |  |  | male | 906 | 24.394 | 20.766 | 28.022 |
| Reject | HMS | Double-blind | female | 3752 | 21.925 | 19.733 | 24.116 |
|  |  |  | male | 14118 | 22.009 | 19.818 | 24.199 |
|  |  | Single-blind | female | 7592 | 21.661 | 20.004 | 23.319 |
|  |  |  | male | 27961 | 21.745 | 20.090 | 23.401 |
|  | LS | Double-blind | female | 475 | 21.226 | 17.620 | 24.833 |
|  |  |  | male | 1028 | 21.310 | 17.705 | 24.916 |
|  |  | Single-blind | female | 1312 | 20.963 | 17.857 | 24.069 |
|  |  |  | male | 3110 | 21.047 | 17.942 | 24.152 |
|  | PS | Double-blind | female | 80 | 21.582 | 19.029 | 24.136 |
|  |  |  | male | 233 | 21.666 | 19.114 | 24.219 |
|  |  | Single-blind | female | 16139 | 21.319 | 20.042 | 22.596 |
|  |  |  | male | 64573 | 21.403 | 20.129 | 22.677 |
|  | SS&E | Double-blind | female | 2628 | 26.938 | 23.312 | 30.564 |
|  |  |  | male | 3451 | 27.022 | 23.396 | 30.647 |
|  |  | Single-blind | female | 638 | 26.674 | 23.046 | 30.303 |
|  |  |  | male | 2418 | 26.758 | 23.130 | 30.386 |

LIWC – Linguistic Inquiry and Word Count software, HMS – Health and Medical Sciences, LS – Life Sciences, PS – Physical sciences, SS&E – Social Sciences and Economics

**Table 10.** LIWC **Authentic** tone mixed model linear regression coefficients and residuals

| Fixed effects | | Standardized estimate | 95% CI | | P |
| --- | --- | --- | --- | --- | --- |
|  | |  | Lower | Upper |  |
|  | (Intercept) | 20.7 | 18.5 | 22.9 | 0.001 |
| Journal’s field of research (reference HMS) | |  |  |  |  |
|  | Life sciences | -0.7 | -4.1 | 2.7 | 0.690 |
|  | Physical sciences | -0.3 | -2.4 | 1.7 | 0.740 |
|  | Social sciences and economics | 5.0 | 1.3 | 8.8 | 0.010 |
| Reviewer recommendation (Reference Accept) | |  |  |  |  |
|  | Minor revision | -1.1 | -1.3 | -0.9 | 0.001 |
|  | Major revision | -1.1 | -1.3 | -0.9 | 0.001 |
|  | Reject | 1.3 | 1.1 | 1.5 | 0.001 |
| Gender: Male | | 0.1 | 0.0 | 0.2 | 0.13 |
| Peer review type: Single blind | | -0.3 | -2.6 | 2.1 | 0.83 |
|  | |  |  |  |  |
|  | |  |  |  |  |
| Random effects | | Standard deviation |  |  |  |
| LIWC Word count | | 1.34 |  |  |  |
| Journal | | 3.48 |  |  |  |
| Article type | | 0.11 |  |  |  |
| Residual | | 15.48 |  |  |  |

LIWC – Linguistic Inquiry and Word Count software, CI – confidence interval, HMS – Health and Medical Sciences

**Table 11.** Examples of review reports with high and low scores for LIWC **Authentic** tone

| **High** |
| --- |
| I believe you are over-reaching in your conclusions. Your meta-analysis only compounds the challenges faced in research of this topic most notably follow-up. I cannot recommend acceptance of this article. |
| I can be very brief on this: everything the authors present is either basic common practice (score plots) or advanced, yet often used in [anonymized]. The research presented here does not warrant publication in this journal. |
| Seems to be a reasonable piece of work. Large number of patients at a [anonymized]. Hasn't been too much on this recently. Nicely written, good conclusions. I would support acceptance. |
| My main concern is the length of the manuscript. There are too many figures. I think some figures can move to the SI. I can't see the QA procedure for the different analytical techniques applied in the current study |
| I have gone over the manuscript. I regret to say that the manuscript received does not contain new understandings; nor does it contain good new observations. The only relatively new results are those presented in Fig. 5 (useful but not significantly important). Consequently, I cannot recommend publication. |
|  |
| **Low** |
| This is an important and carefully crafted paper. It has practical importance, as the methods of producing [anonymized] are relatively unused but appear to have great potential. This paper provides meaningful and simply employed analytical methods for designing [anonymized]. It is a valuable contribution. It would have been worthwhile if the authors would have more fully discussed the potential role that [anonymized] and a [anonymized] plays in this method. |
| It is an interesting topic, and the approach applied seems appropriate. So, there is a good chance that with the required corrections this will be a valuable paper. However, the paper is very poorly written, and must be improved significantly. Comments and suggestions are given in the attached review. |
| Suggestions/Comments. 1. Did the author try and contact the authors of RCT trials to get patient level. This will strengthen the meta-analysis results and thereby come to conclusion. 2. Need to mention about reporting of RCT. Need to follow PRISMA reporting guidelines. 3. Need to include follow chart of data extraction |
| The authors should comment the computational efficiency of the proposed technique. The authors should use a statistical test for the comparisons between the techniques in their experiments A larger [anonymized] data set should be also used for testing the scalability of the proposed technique. |
| This is an interesting paper from a renowned group of investigators. Despite the short period of follow-up, the authors could observe some incident cases of [anonymized] in this cohort. Some comments: 1. Were there any chances of misclassification of [anonymized]. If any, how these could influence the results? 2. Table 4 shows the odds ratios for multivariable analyses. It would be interesting to see the values of all variables in each model. 3. It would be interesting to add comments on how the results of this type of research would translate into daily medical practice and how the results could be generalized to larger [anonymized] populations. |
