# Supplementary material for: Large-scale language analysis of peer review reports
Source: eLife. 2020 Jul 17;9:e53249. doi: 10.7554/eLife.53249 (PMC7390598; doi:10.7554/eLife.53249)
Supplement: Supplementary file 5. [file elife-53249-supp5.docx]

**Buljan et al. 2020 eLife 9:e53249. Supplementary file 5**

**Sentiment/LIWC emotional tone (figure 3A): summary data, mixed model linear regression coefficients and residuals, and examples of reports with high and low scores for sentiment (LIWC emotional tone)**

**Table 12.** Average LIWC **Emotional** tone levels in review reports per reviewer recommendation, journal’s field of research, type of peer review type and reviewer’s gender (range 0-100)

| **Reviewer recommendation** | **Journal’s field of research** | **Peer review type** | **Reviewer gender** | **N** | **Predicted mean** | **Lower 95% CI** | **Upper 95% CI** |
| --- | --- | --- | --- | --- | --- | --- | --- |
| Accept | HMS | Double-blind | female | 729 | 61.572 | 58.843 | 64.300 |
|  |  |  | male | 3044 | 60.765 | 58.038 | 63.492 |
|  |  | Single-blind | female | 1526 | 61.499 | 59.325 | 63.672 |
|  |  |  | male | 5113 | 60.692 | 58.521 | 62.863 |
|  | LS | Double-blind | female | 89 | 52.461 | 48.200 | 56.722 |
|  |  |  | male | 255 | 51.654 | 47.394 | 55.915 |
|  |  | Single-blind | female | 201 | 52.388 | 48.680 | 56.095 |
|  |  |  | male | 478 | 51.581 | 47.875 | 55.287 |
|  | PS | Double-blind | female | 16 | 61.998 | 58.880 | 65.115 |
|  |  |  | male | 92 | 61.191 | 58.075 | 64.306 |
|  |  | Single-blind | female | 2669 | 61.924 | 60.120 | 63.729 |
|  |  |  | male | 11591 | 61.118 | 59.317 | 62.918 |
|  | SS&E | Double-blind | female | 221 | 69.039 | 64.760 | 73.319 |
|  |  |  | male | 193 | 68.233 | 63.954 | 72.512 |
|  |  | Single-blind | female | 20 | 68.966 | 64.681 | 73.252 |
|  |  |  | male | 150 | 68.160 | 63.875 | 72.444 |
| Minor revision | HMS | Double-blind | female | 737 | 52.154 | 49.439 | 54.869 |
|  |  |  | male | 2151 | 51.347 | 48.634 | 54.061 |
|  |  | Single-blind | female | 7983 | 52.081 | 49.925 | 54.237 |
|  |  |  | male | 23822 | 51.274 | 49.121 | 53.427 |
|  | LS | Double-blind | female | 827 | 43.043 | 38.791 | 47.296 |
|  |  |  | male | 1532 | 42.237 | 37.985 | 46.488 |
|  |  | Single-blind | female | 1924 | 42.970 | 39.273 | 46.667 |
|  |  |  | male | 3925 | 42.164 | 38.468 | 45.859 |
|  | PS | Double-blind | female | 102 | 52.580 | 49.474 | 55.686 |
|  |  |  | male | 251 | 51.773 | 48.669 | 54.877 |
|  |  | Single-blind | female | 24506 | 52.507 | 50.724 | 54.290 |
|  |  |  | male | 84040 | 51.700 | 49.921 | 53.479 |
|  | SS&E | Double-blind | female | 3939 | 59.622 | 55.351 | 63.893 |
|  |  |  | male | 3902 | 58.815 | 54.545 | 63.085 |
|  |  | Single-blind | female | 447 | 59.549 | 55.273 | 63.825 |
|  |  |  | male | 1608 | 58.742 | 54.467 | 63.017 |
| Major revision | HMS | Double-blind | female | 3242 | 46.556 | 43.841 | 49.271 |
|  |  |  | male | 7756 | 45.749 | 43.036 | 48.462 |
|  |  | Single-blind | female | 10327 | 46.483 | 44.328 | 48.638 |
|  |  |  | male | 26235 | 45.676 | 43.524 | 47.828 |
|  | LS | Double-blind | female | 579 | 37.445 | 33.194 | 41.697 |
|  |  |  | male | 1175 | 36.639 | 32.388 | 40.890 |
|  |  | Single-blind | female | 1379 | 37.372 | 33.676 | 41.068 |
|  |  |  | male | 2855 | 36.566 | 32.871 | 40.260 |
|  | PS | Double-blind | female | 60 | 46.982 | 43.877 | 50.087 |
|  |  |  | male | 196 | 46.175 | 43.072 | 49.278 |
|  |  | Single-blind | female | 16225 | 46.909 | 45.128 | 48.690 |
|  |  |  | male | 59842 | 46.102 | 44.325 | 47.879 |
|  | SS&E | Double-blind | female | 2017 | 54.024 | 49.754 | 58.294 |
|  |  |  | male | 1852 | 53.217 | 48.948 | 57.486 |
|  |  | Single-blind | female | 212 | 53.951 | 49.676 | 58.226 |
|  |  |  | male | 906 | 53.144 | 48.870 | 57.418 |
| Reject | HMS | Double-blind | female | 3752 | 38.552 | 35.837 | 41.267 |
|  |  |  | male | 14118 | 37.745 | 35.032 | 40.458 |
|  |  | Single-blind | female | 7592 | 38.479 | 36.323 | 40.634 |
|  |  |  | male | 27961 | 37.672 | 35.520 | 39.825 |
|  | LS | Double-blind | female | 475 | 29.441 | 25.189 | 33.694 |
|  |  |  | male | 1028 | 28.635 | 24.383 | 32.886 |
|  |  | Single-blind | female | 1312 | 29.368 | 25.672 | 33.065 |
|  |  |  | male | 3110 | 28.562 | 24.866 | 32.257 |
|  | PS | Double-blind | female | 80 | 38.978 | 35.872 | 42.084 |
|  |  |  | male | 233 | 38.171 | 35.068 | 41.275 |
|  |  | Single-blind | female | 16139 | 38.905 | 37.122 | 40.687 |
|  |  |  | male | 64573 | 38.098 | 36.320 | 39.876 |
|  | SS&E | Double-blind | female | 2628 | 46.020 | 41.750 | 50.290 |
|  |  |  | male | 3451 | 45.213 | 40.944 | 49.482 |
|  |  | Single-blind | female | 638 | 45.947 | 41.671 | 50.222 |
|  |  |  | male | 2418 | 45.140 | 40.866 | 49.414 |

LIWC – Linguistic Inquiry and Word Count software, HMS – Health and Medical Sciences, LS – Life Sciences, PS – Physical sciences, SS&E – Social Sciences and Economics

**Table 13.** LIWC **Emotional** Tone mixed model linear regression coefficients and residuals

| Fixed effects | | Standardized estimate | 95% CI | | P |
| --- | --- | --- | --- | --- | --- |
|  | |  | Lower | Upper |  |
|  | (Intercept) | 61.57 | 58.84 | 64.3 | <0.001 |
| Journal’s field of research (reference HMS) | |  |  |  |  |
|  | Life sciences | -9.11 | -13.01 | -5.22 | <0.001 |
|  | Physical sciences | 0.43 | -1.88 | 2.73 | 0.720 |
|  | Social sciences and economics | 7.47 | 3.18 | 11.75 | <0.001 |
| Reviewer recommendation (Reference Accept) | |  |  |  |  |
|  | Minor revision | -9.42 | -9.74 | -9.09 | <0.001 |
|  | Major revision | -15.02 | -15.35 | -14.68 | <0.001 |
|  | Reject | -23.02 | -23.35 | -22.69 | <0.001 |
| Gender: Male | | -0.81 | -0.97 | -0.64 | <0.001 |
| Peer review type: Single blind | | -0.07 | -2.79 | 2.65 | 0.96 |
|  | |  |  |  |  |
|  | |  |  |  |  |
| Random effects | | Standard deviation |  |  |  |
| LIWC Word count | | 4.37 |  |  |  |
| Journal | | 3.97 |  |  |  |
| Article type | | 0.73 |  |  |  |
| Residual | | 23.37 |  |  |  |

LIWC – Linguistic Inquiry and Word Count software, CI – confidence interval, HMS – Health and Medical Sciences

**Table 14.** Examples of review reports with high and low scores for LIWC **Emotional** tone

| **High** |
| --- |
| The supports are very important for the [anonymized] and the authors presented good results. Only one question: Could the authors give some suggestions to reduce the supports for saving the materials and time? |
| Thank you for this very interesting paper on this important topic. It fulfils the criteria of good science practice and also offers new findings which may help scientists and professionals in diagnostics and treatment of [anonymized]. |
| On reviewing the submission, I found it’s a part of very useful and applicable research in this area. The research seems to be precise and informational with good references. The author has really done hard work and in my viewpoint, the submission in acceptable in its present form. The research can also be presented at some good workshop/conference. In nutshell, I appreciate the paper and we can go ahead with present form. |
| I agree with the authors of the importance of the information provided within this article. Thus, although long in length, I see no reason to shorten the article. It is well-written on a topic of interest to our readers. |
| This is a well written paper reporting on an important topic. I would like the authors to include a paragraph in the discussion section of the limitations and strengths of their study. Also, it will be nice to see a picture of the experimental set up. |
| Decent work in the field of [anonymized] I would recommend for a publication. Please keep up the good work and keep investigating further this area is very deserving of exploration. Definitely needs further investigation it’s a move in right direction. Extremely relevant to this Journal. Strong recommendation. |
| **Low** |
| This a methodologically simple study. Indeed, the authors have retrospectively addressed the predictors of [anonymized] in patients undergoing [anonymized] test. The statistical analysis is straightforward. Unfortunately, the clinical relevance of this study is limited, because [anonymized] is not the most important outcome of [anonymized] test. |
| After a careful study of the manuscript I must recommend paper rejection. The main problem of the study is usage of unreliable data. Namely, identification (and afterwards quantification) of [anonymized] based only on [anonymized] reported in literature is not proper and suitable. |
| I would like to know how she rules out [anonymized] patients even with low [anonymized]. How many eye were excluded because of bad image quality due to shaking of the head. this is not explained or mentioned. |
| The authors use for their experiments [anonymized] that describe supervised (classification) problems The authors should clearly describe how these [anonymized] are transformed to weakly [anonymized] and how cross-validation is applied in that case. |
| Presentation is poor, novelty looks low. Just reformat a list of [anonymized] into [anonymized]. No proof for a number of 'rewritten form’. Simulation is not clear. Only one example for comparison is not enough. |
