# Supplementary material for: Large-scale language analysis of peer review reports
Source: eLife. 2020 Jul 17;9:e53249. doi: 10.7554/eLife.53249 (PMC7390598; doi:10.7554/eLife.53249)
Supplement: Supplementary file 6. [file elife-53249-supp6.docx]

**Buljan et al. 2020 eLife 9:e53249. Supplementary file 6**

**Sentiment/SentimentR score (figure 3B): summary data, mixed model linear regression coefficients and residuals, and examples of reports with high and low scores for sentiment (SentimentR scores)**

**Table 15.** Average **SentimentR** levels in review reports per reviewer recommendation, journal’s field of research, type of peer review type and reviewer’s gender (range -1 to +1)

| **Reviewer recommendation** | **Journal’s field of research** | **Peer review type** | **Reviewer gender** | **N** | **Predicted mean** | **Lower 95% CI** | **Upper95% CI** |
| --- | --- | --- | --- | --- | --- | --- | --- |
| Accept | HMS | Double-blind | female | 729 | 0.198 | 0.187 | 0.209 |
|  |  |  | male | 3044 | 0.190 | 0.179 | 0.201 |
|  |  | Single-blind | female | 1526 | 0.200 | 0.191 | 0.208 |
|  |  |  | male | 5113 | 0.191 | 0.183 | 0.200 |
|  | LS | Double-blind | female | 89 | 0.181 | 0.163 | 0.199 |
|  |  |  | male | 255 | 0.173 | 0.155 | 0.191 |
|  |  | Single-blind | female | 201 | 0.183 | 0.167 | 0.198 |
|  |  |  | male | 478 | 0.174 | 0.159 | 0.190 |
|  | PS | Double-blind | female | 16 | 0.218 | 0.206 | 0.231 |
|  |  |  | male | 92 | 0.210 | 0.197 | 0.223 |
|  |  | Single-blind | female | 2669 | 0.220 | 0.213 | 0.226 |
|  |  |  | male | 11591 | 0.212 | 0.205 | 0.218 |
|  | SS&E | Double-blind | female | 221 | 0.243 | 0.225 | 0.261 |
|  |  |  | male | 193 | 0.235 | 0.217 | 0.252 |
|  |  | Single-blind | female | 20 | 0.245 | 0.227 | 0.262 |
|  |  |  | male | 150 | 0.236 | 0.218 | 0.254 |
| Minor revision | HMS | Double-blind | female | 737 | 0.112 | 0.101 | 0.123 |
|  |  |  | male | 2151 | 0.103 | 0.092 | 0.114 |
|  |  | Single-blind | female | 7983 | 0.113 | 0.105 | 0.121 |
|  |  |  | male | 23822 | 0.105 | 0.096 | 0.113 |
|  | LS | Double-blind | female | 827 | 0.095 | 0.077 | 0.112 |
|  |  |  | male | 1532 | 0.086 | 0.068 | 0.104 |
|  |  | Single-blind | female | 1924 | 0.096 | 0.081 | 0.111 |
|  |  |  | male | 3925 | 0.088 | 0.072 | 0.103 |
|  | PS | Double-blind | female | 102 | 0.132 | 0.119 | 0.144 |
|  |  |  | male | 251 | 0.123 | 0.111 | 0.136 |
|  |  | Single-blind | female | 24506 | 0.133 | 0.127 | 0.140 |
|  |  |  | male | 84040 | 0.125 | 0.119 | 0.131 |
|  | SS&E | Double-blind | female | 3939 | 0.156 | 0.138 | 0.174 |
|  |  |  | male | 3902 | 0.148 | 0.130 | 0.166 |
|  |  | Single-blind | female | 447 | 0.158 | 0.140 | 0.176 |
|  |  |  | male | 1608 | 0.149 | 0.132 | 0.167 |
| Major revision | HMS | Double-blind | female | 3242 | 0.079 | 0.068 | 0.090 |
|  |  |  | male | 7756 | 0.071 | 0.060 | 0.082 |
|  |  | Single-blind | female | 10327 | 0.081 | 0.073 | 0.089 |
|  |  |  | male | 26235 | 0.072 | 0.064 | 0.081 |
|  | LS | Double-blind | female | 579 | 0.062 | 0.044 | 0.080 |
|  |  |  | male | 1175 | 0.054 | 0.036 | 0.072 |
|  |  | Single-blind | female | 1379 | 0.064 | 0.048 | 0.079 |
|  |  |  | male | 2855 | 0.055 | 0.040 | 0.071 |
|  | PS | Double-blind | female | 60 | 0.100 | 0.087 | 0.112 |
|  |  |  | male | 196 | 0.091 | 0.078 | 0.104 |
|  |  | Single-blind | female | 16225 | 0.101 | 0.095 | 0.107 |
|  |  |  | male | 59842 | 0.093 | 0.086 | 0.099 |
|  | SS&E | Double-blind | female | 2017 | 0.124 | 0.106 | 0.142 |
|  |  |  | male | 1852 | 0.116 | 0.098 | 0.134 |
|  |  | Single-blind | female | 212 | 0.126 | 0.108 | 0.144 |
|  |  |  | male | 906 | 0.117 | 0.099 | 0.135 |
| Reject | HMS | Double-blind | female | 3752 | 0.031 | 0.021 | 0.042 |
|  |  |  | male | 14118 | 0.023 | 0.012 | 0.034 |
|  |  | Single-blind | female | 7592 | 0.033 | 0.025 | 0.041 |
|  |  |  | male | 27961 | 0.025 | 0.016 | 0.033 |
|  | LS | Double-blind | female | 475 | 0.014 | -0.003 | 0.032 |
|  |  |  | male | 1028 | 0.006 | -0.012 | 0.024 |
|  |  | Single-blind | female | 1312 | 0.016 | 0.001 | 0.031 |
|  |  |  | male | 3110 | 0.007 | -0.008 | 0.023 |
|  | PS | Double-blind | female | 80 | 0.052 | 0.039 | 0.064 |
|  |  |  | male | 233 | 0.043 | 0.030 | 0.056 |
|  |  | Single-blind | female | 16139 | 0.053 | 0.047 | 0.060 |
|  |  |  | male | 64573 | 0.045 | 0.038 | 0.051 |
|  | SS&E | Double-blind | female | 2628 | 0.076 | 0.058 | 0.094 |
|  |  |  | male | 3451 | 0.068 | 0.050 | 0.086 |
|  |  | Single-blind | female | 638 | 0.078 | 0.060 | 0.096 |
|  |  |  | male | 2418 | 0.069 | 0.051 | 0.087 |

HMS – Health and Medical Sciences, LS – Life Sciences, PS – Physical sciences, SS&E – Social Sciences and Economics

**Table 16.** **SentimentR** mixed model linear regression coefficients and residuals

| Fixed effects | | Standardized estimate | 95% CI | | P |
| --- | --- | --- | --- | --- | --- |
|  | |  | Lower | Upper |  |
|  | (Intercept) | 0.20 | 0.19 | 0.2 | <0.001 |
| Journal’s field of research (reference HMS) | |  |  |  |  |
|  | Life sciences | -0.02 | -0.03 | 0.00 | 0.050 |
|  | Physical sciences | 0.02 | 0.01 | 0.03 | <0.001 |
|  | Social sciences and economics | 0.04 | 0.03 | 0.06 | <0.001 |
| Reviewer recommendation (Reference Accept) | |  |  |  |  |
|  | Minor revision | -0.09 | -0.09 | -0.09 | <0.001 |
|  | Major revision | -0.12 | -0.12 | -0.12 | <0.001 |
|  | Reject | -0.17 | -0.17 | -0.17 | <0.001 |
| Gender: Male | | -0.01 | -0.01 | -0.01 | <0.001 |
| Peer review type: Single blind | | 0.00 | -0.01 | 0.01 | 0.800 |
|  | |  |  |  |  |
|  | |  |  |  |  |
| Random effects | | Standard deviation |  |  |  |
| LIWC Word count | | 0.01 |  |  |  |
| Journal | | 0.02 |  |  |  |
| Article type | | 0.00 |  |  |  |
| Residual | | 0.11 |  |  |  |

LIWC – Linguistic Inquiry and Word Count software, CI – confidence interval, HMS – Health and Medical Sciences

**Table 17.** Examples of review reports with high and low scores for **SentimentR**

| **High** |
| --- |
| Very good article very well presented and justified. This article encourages the young surgeon to take into serious consideration the [anonymized] in [anonymized] and have better clinical outcomes. |
| Your work is very interesting; in my opinion the most important is the use and knowledge to develop a [anonymized] to model your system, the expert knowledge is always important to improve a soft [anonymized] system. |
| The topic is interesting, the work structure and the scientific content of the paper is good, the academic level of the paper is good, the conclusions are justified. Overall, this paper is of a good quality and well-written, the recommendation is to accept this paper for publication. |
| This is a well performed study. The text is clearly written, the experiments appear to be solid and the results are clearly presented. The discussion as well as the conclusion are based on solid references and describe the results very well. Overall: all well written ad solid study. |
| The article is very well organized and reports important results. The authors have extensive knowledge on [anonymized] analysis and, therefore, the discussion is very exhaustive I think the article should be accepted in the present form. |
|  |
| **Low** |
| The purpose of the paper is not clear for me. The overall re-discussion is required, especially, the part of [anonymized] is insufficient. You are correcting repeatedly but cannot find out scientific importance I strongly recommend you of submitting another chance. |
| The findings may be valid, but I'm afraid that the English is so poor that I can't sensibly recommend acceptance. Getting a native English speaker with a medical background to review the paper for you would be sensible |
| The experiments lead to the conclusion that the proposed method is very efficient but there is too much work on the in-depth analysis of this method to understand why it is working. |
| I am very sorry, but I cannot see the novelty of this paper, nor the relevance. One of my criteria for recommendations is whether I learned something new from the paper, that was not the case this time. |
| The description and discussion of the results is not sufficient. Sometimes the logic is not clear or orderless, and a basic description of the rationale in behind the results is lacking and it would be very much up to the reader to speculate on that. |
