# Supplementary material for: Large-scale language analysis of peer review reports
Source: eLife. 2020 Jul 17;9:e53249. doi: 10.7554/eLife.53249 (PMC7390598; doi:10.7554/eLife.53249)
Supplement: Supplementary file 7. [file elife-53249-supp7.docx]

**Buljan et al. 2020 eLife 9:e53249. Supplementary file 7**

**Sentiment/Stanford CoreNLP score (figure 3C): summary data, mixed model linear regression coefficients and residuals, and examples of reports with high and low scores for sentiment (Stanford CoreNLP score)**

**Table 18.** Average **CoreNLP Polarity Sentiment** in review reports per reviewer recommendation, journal’s field of research, type of peer review type and reviewer’s gender (range 0-4)

| **Reviewer recommendation** | **Journal’s field of research** | **Peer review type** | **Reviewer gender** | **N** | **Predicted mean** | **Lower 95% CI** | **Upper95% CI** |
| --- | --- | --- | --- | --- | --- | --- | --- |
| Accept | HMS | Double-blind | female | 729 | 1.839 | 1.820 | 1.859 |
|  |  |  | male | 3044 | 1.829 | 1.810 | 1.848 |
|  |  | Single-blind | female | 1526 | 1.834 | 1.818 | 1.850 |
|  |  |  | male | 5113 | 1.824 | 1.808 | 1.839 |
|  | LS | Double-blind | female | 89 | 1.814 | 1.784 | 1.843 |
|  |  |  | male | 255 | 1.803 | 1.774 | 1.833 |
|  |  | Single-blind | female | 201 | 1.808 | 1.783 | 1.834 |
|  |  |  | male | 478 | 1.798 | 1.772 | 1.824 |
|  | PS | Double-blind | female | 16 | 1.856 | 1.834 | 1.878 |
|  |  |  | male | 92 | 1.846 | 1.824 | 1.868 |
|  |  | Single-blind | female | 2669 | 1.851 | 1.837 | 1.864 |
|  |  |  | male | 11591 | 1.840 | 1.827 | 1.854 |
|  | SS&E | Double-blind | female | 221 | 1.891 | 1.862 | 1.921 |
|  |  |  | male | 193 | 1.881 | 1.852 | 1.910 |
|  |  | Single-blind | female | 20 | 1.886 | 1.857 | 1.915 |
|  |  |  | male | 150 | 1.876 | 1.846 | 1.905 |
| Minor revision | HMS | Double-blind | female | 737 | 1.652 | 1.632 | 1.671 |
|  |  |  | male | 2151 | 1.641 | 1.622 | 1.661 |
|  |  | Single-blind | female | 7983 | 1.646 | 1.631 | 1.662 |
|  |  |  | male | 23822 | 1.636 | 1.620 | 1.652 |
|  | LS | Double-blind | female | 827 | 1.626 | 1.597 | 1.656 |
|  |  |  | male | 1532 | 1.616 | 1.586 | 1.645 |
|  |  | Single-blind | female | 1924 | 1.621 | 1.595 | 1.647 |
|  |  |  | male | 3925 | 1.610 | 1.584 | 1.636 |
|  | PS | Double-blind | female | 102 | 1.669 | 1.647 | 1.691 |
|  |  |  | male | 251 | 1.658 | 1.636 | 1.680 |
|  |  | Single-blind | female | 24506 | 1.663 | 1.650 | 1.677 |
|  |  |  | male | 84040 | 1.653 | 1.639 | 1.666 |
|  | SS&E | Double-blind | female | 3939 | 1.704 | 1.675 | 1.733 |
|  |  |  | male | 3902 | 1.693 | 1.664 | 1.722 |
|  |  | Single-blind | female | 447 | 1.698 | 1.669 | 1.728 |
|  |  |  | male | 1608 | 1.688 | 1.659 | 1.717 |
| Major revision | HMS | Double-blind | female | 3242 | 1.588 | 1.569 | 1.608 |
|  |  |  | male | 7756 | 1.578 | 1.558 | 1.597 |
|  |  | Single-blind | female | 10327 | 1.583 | 1.567 | 1.599 |
|  |  |  | male | 26235 | 1.572 | 1.556 | 1.588 |
|  | LS | Double-blind | female | 579 | 1.562 | 1.533 | 1.592 |
|  |  |  | male | 1175 | 1.552 | 1.523 | 1.581 |
|  |  | Single-blind | female | 1379 | 1.557 | 1.531 | 1.583 |
|  |  |  | male | 2855 | 1.547 | 1.521 | 1.573 |
|  | PS | Double-blind | female | 60 | 1.605 | 1.583 | 1.627 |
|  |  |  | male | 196 | 1.594 | 1.573 | 1.616 |
|  |  | Single-blind | female | 16225 | 1.600 | 1.586 | 1.613 |
|  |  |  | male | 59842 | 1.589 | 1.575 | 1.603 |
|  | SS&E | Double-blind | female | 2017 | 1.640 | 1.611 | 1.669 |
|  |  |  | male | 1852 | 1.630 | 1.601 | 1.659 |
|  |  | Single-blind | female | 212 | 1.635 | 1.606 | 1.664 |
|  |  |  | male | 906 | 1.624 | 1.595 | 1.654 |
| Reject | HMS | Double-blind | female | 3752 | 1.499 | 1.480 | 1.518 |
|  |  |  | male | 14118 | 1.489 | 1.469 | 1.508 |
|  |  | Single-blind | female | 7592 | 1.494 | 1.478 | 1.510 |
|  |  |  | male | 27961 | 1.483 | 1.467 | 1.499 |
|  | LS | Double-blind | female | 475 | 1.473 | 1.444 | 1.503 |
|  |  |  | male | 1028 | 1.463 | 1.434 | 1.492 |
|  |  | Single-blind | female | 1312 | 1.468 | 1.442 | 1.494 |
|  |  |  | male | 3110 | 1.458 | 1.432 | 1.483 |
|  | PS | Double-blind | female | 80 | 1.516 | 1.494 | 1.538 |
|  |  |  | male | 233 | 1.505 | 1.484 | 1.527 |
|  |  | Single-blind | female | 16139 | 1.510 | 1.497 | 1.524 |
|  |  |  | male | 64573 | 1.500 | 1.486 | 1.514 |
|  | SS&E | Double-blind | female | 2628 | 1.551 | 1.522 | 1.580 |
|  |  |  | male | 3451 | 1.541 | 1.512 | 1.570 |
|  |  | Single-blind | female | 638 | 1.546 | 1.517 | 1.575 |
|  |  |  | male | 2418 | 1.535 | 1.506 | 1.564 |

NLP – natural language programming, HMS – Health and Medical Sciences, LS – Life Sciences, PS – Physical sciences, SS&E – Social Sciences and Economics

**Table 19.** Core NLP **Sentiment** mean mixed model linear regression coefficients and residuals

| Fixed effects | | Standardized estimate | 95% CI | | P |
| --- | --- | --- | --- | --- | --- |
|  | |  | Lower | Upper |  |
|  | (Intercept) | 1.84 | 1.82 | 184.68 | <0.001 |
| Journal’s field of research (reference HMS) | |  |  |  |  |
|  | Life sciences | -0.03 | -0.05 | 0.00 | 0.06 |
|  | Physical sciences | 0.02 | 0.00 | 0.03 | 0.04 |
|  | Social sciences and economics | 0.05 | 0.02 | 0.08 | <0.001 |
| Reviewer recommendation (Reference Accept) | |  |  |  |  |
|  | Minor revision | -0.19 | -0.19 | -0.18 | <0.001 |
|  | Major revision | -0.25 | -0.25 | -0.25 | <0.001 |
|  | Reject | -0.34 | -0.34 | -0.34 | <0.001 |
| Gender: Male | | -0.01 | -0.01 | -0.01 | <0.001 |
| Peer review type: Single blind | | -0.01 | -0.02 | 0.01 | 0.560 |
|  | |  |  |  |  |
|  | |  |  |  |  |
| Random effects | | Standard deviation |  |  |  |
| LIWC Word count | | 0.06 |  |  |  |
| Journal | | 0.03 |  |  |  |
| Article type | | 0.01 |  |  |  |
| Residual | | 0.23 |  |  |  |

LIWC – Linguistic Inquiry and Word Count software, CI – confidence interval, HMS – Health and Medical Sciences

**Table 20.** Examples of review reports with high and low scores for **Stanford CoreNLP sentiment**

| **High** |
| --- |
| The topic is very interesting, the work is sound, clearly and convincingly presented. The mathematical model is complex, and the authors have the merit of mastering it into defining and finding optimal working conditions for [anonymized]. |
| The manuscript is well written, the abstract is succinct and accurately reflex content, the introduction is adequate, the methods are sound but compounded by possible recall bias, figures and table are well done, the references are relevant and adequate. |
| The topic is interesting, the work structure and the scientific content of the paper is good, the academic level of the paper is good, the conclusions are justified. Overall, this paper is of a good quality and well-written, the recommendation is to accept this paper for publication. |
| The article is gorgeous. Excellent contribution to his journal Literature review is update; moreover, results and discussion as well as the conclusion chapters are very good indeed Article presents a very good format. |
| the manuscript is written in in a very scientific style and according to the journal style it is a very good and new method to [anonymized] I recommend to publish the manuscript. |
|  |
| **Low** |
| The manuscript is rejected: 1) the specific information is of limited value, as the literature indicates other such studies: 2) analysis of data was limited; 3) there are no Research Highlights |
| This is an extremely poorly written manuscript that is very difficult to follow and understand. The authors may have something interesting to report; however, the manuscript must be rewritten in good English. |
| This paper is too simple and too short; it only provided the basic knowledge about [anonymized], there is little innovation, and it cannot be accepted for this high-level journal. |
| Novelty of the work is not convincingly covered; the use of the [anonymized] is questionable in the context and seemingly completely ignores specifics of the alternative [anonymized] method. |
| In my opinion the article does not convey enough new information for being published in a journal with such impact factor Indeed, the methodologies applied are standard, the hypothesis is not innovative and the results lack proper statistical analysis and interpretation |
