# Supplementary material for: Large-scale language analysis of peer review reports
Source: eLife. 2020 Jul 17;9:e53249. doi: 10.7554/eLife.53249 (PMC7390598; doi:10.7554/eLife.53249)
Supplement: Supplementary file 8. [file elife-53249-supp8.docx]

**Buljan et al. 2020 eLife 9:e53249. Supplementary file 8**

**Ten most frequent words found in peer review reports for general morality and the five moral foundation variables**

**Table 21.** Ten most frequent words found in review reports for Morality-General across journal’s fields of research

| **Morality-General (HMS, total=167)** | | **Morality-General (LS, total=84)** | | **Morality-General (PS, total=235)** | | **Morality-General (SS&E, total=172)** | |
| --- | --- | --- | --- | --- | --- | --- | --- |
| **Word** | **Freq.** | **Word** | **Freq** | **Word** | **Freq** | **Word** | **Freq** |
| values | 43476 | correct | 6853 | values | 136989 | good | 16779 |
| value | 36829 | values | 5492 | good | 114625 | value | 12762 |
| good | 33131 | good | 4673 | value | 96966 | values | 6719 |
| correct | 24198 | value | 4074 | correct | 84836 | correct | 4534 |
| worth | 5477 | wrong | 1375 | wrong | 26957 | worth | 2929 |
| wrong | 4793 | proper | 1326 | proper | 21789 | ethical | 2175 |
| proper | 4703 | worth | 1093 | worth | 14676 | wrong | 2024 |
| ethical | 2787 | integrity | 556 | bad | 8316 | moral | 1744 |
| ideal | 2318 | bad | 400 | principle | 7323 | bad | 1717 |
| worthwhile | 2205 | principle | 363 | worthy | 5830 | legal | 1457 |

HMS – Health and Medical Sciences, LS – Life Sciences, PS – Physical sciences, SS&E – Social Sciences and Economics

**Table 22.** Ten most frequent words found in review reports for Care-Virtue across journal’s fields of research

| **Care-Virtue (HMS, total=181)** | | **Care-Virtue (LS, total=92)** | | **Care-Virtue (PS, total=230)** | | **Care-Virtue (SS&E, total=230)** | |
| --- | --- | --- | --- | --- | --- | --- | --- |
| **Word** | **Freq.** | **Word** | **Freq** | **Word** | **Freq** | **Word** | **Freq** |
| care | 31511 | benefit | 1772 | benefit | 18022 | care | 18599 |
| benefit | 16307 | protective | 1219 | protection | 9332 | benefit | 6025 |
| safety | 6099 | protection | 777 | benefits | 9007 | benefits | 3368 |
| benefits | 5333 | care | 593 | care | 7415 | safety | 1197 |
| protective | 4347 | protect | 302 | security | 7357 | security | 1027 |
| safe | 2812 | benefits | 281 | protected | 5474 | protection | 1001 |
| protection | 2711 | sympathetic | 264 | safety | 4497 | protective | 659 |
| sympathetic | 1105 | protects | 195 | protective | 2511 | safe | 566 |
| protect | 965 | defense | 182 | safe | 2460 | caring | 432 |
| protects | 648 | empathy | 182 | protect | 2020 | protect | 371 |

HMS – Health and Medical Sciences, LS – Life Sciences, PS – Physical sciences, SS&E – Social Sciences and Economics

**Table 23.** ten most frequent words found in review reports for Care-Vice across journal’s fields of research

| **Care-Vice (HMS, total=154)** | | **Care-Vice (LS, total=90)** | | **Care-Vice (PS, total=256)** | | **Care-Vice (SS&E, total=230)** | |
| --- | --- | --- | --- | --- | --- | --- | --- |
| **Word** | **Freq** | **Word** | **Freq** | **Word** | **Freq** | **Word** | **Freq** |
| damage | 6227 | damage | 2582 | damage | 12991 | violence | 3302 |
| wound | 3422 | damaged | 493 | suffers | 4874 | war | 1114 |
| abuse | 2152 | suffers | 397 | attack | 3402 | suffers | 835 |
| violence | 1821 | killing | 229 | suffer | 2087 | abuse | 815 |
| suffers | 1747 | suffer | 206 | attacks | 2046 | harm | 746 |
| harm | 1181 | wound | 190 | endangered | 1927 | suffer | 645 |
| wounds | 1167 | killed | 163 | exploited | 1719 | suffering | 535 |
| suffer | 1082 | suffering | 161 | damaged | 1647 | harmful | 398 |
| suffering | 976 | abuse | 160 | harmful | 1590 | exploit | 376 |
| suffered | 829 | detrimental | 139 | harmonic | 1574 | detrimental | 312 |

HMS – Health and Medical Sciences, LS – Life Sciences, PS – Physical sciences, SS&E – Social Sciences and Economics

**Table 24.** Ten most frequent words found in review reports for Fairness-Virtue across journal’s fields of research

| **Fairness-Virtue (HMS, total=80)** | | **Fairness-Virtue (LS, total=58)** | | **Fairness-Virtue (PS, total=144)** | | **Fairness-Virtue (SS&E, total=82)** | |
| --- | --- | --- | --- | --- | --- | --- | --- |
| **Word** | **Freq.** | **Word** | **Freq** | **Word** | **Freq** | **Word** | **Freq** |
| reasonable | 7479 | justified | 1091 | constant | 23049 | justification | 4166 |
| justification | 6937 | reasonable | 974 | justified | 18738 | equity | 2414 |
| balance | 6211 | justification | 844 | reasonable | 16860 | reasonable | 1971 |
| justified | 5062 | homologous | 592 | justification | 15788 | equal | 1901 |
| equal | 4240 | equal | 585 | equal | 11124 | justified | 1901 |
| fairly | 4034 | fairly | 547 | balance | 10082 | fairly | 1608 |
| constant | 3435 | equivalent | 529 | equivalent | 8613 | rights | 1478 |
| equivalent | 3327 | equally | 463 | fairly | 7259 | constant | 1359 |
| equally | 2288 | constant | 424 | fair | 6372 | justice | 1284 |
| fair | 2097 | balance | 316 | equally | 3510 | equally | 1199 |

HMS – Health and Medical Sciences, LS – Life Sciences, PS – Physical sciences, SS&E – Social Sciences and Economics

**Table 25.** Ten most frequent words found in review reports for Fairness-Vice across journal’s fields of research

| **Fairness-Vice (HMS, total=105)** | | **Fairness-Vice (LS, total=56)** | | **Fairness-Vice (PS, total=121)** | | **Fairness-Vice (SS&E, total=72)** | |
| --- | --- | --- | --- | --- | --- | --- | --- |
| **Word** | **Freq.** | **Word** | **Freq** | **Word** | **Freq** | **Word** | **Freq** |
| bias | 18359 | bias | 1872 | bias | 11273 | bias | 6495 |
| excluded | 14363 | excluded | 1031 | excluded | 4600 | discrimination | 2998 |
| exclusion | 9104 | exclude | 803 | biased | 3830 | preference | 2355 |
| exclude | 4306 | discrimination | 694 | exclude | 3228 | excluded | 1777 |
| biased | 2628 | biased | 543 | exclusion | 3218 | biased | 1563 |
| excluding | 2240 | exclusion | 490 | preference | 2611 | exclusion | 1509 |
| biases | 1573 | discriminate | 345 | discrimination | 1726 | biases | 1230 |
| preference | 1572 | biases | 340 | excluding | 1718 | segregation | 923 |
| discrimination | 1329 | preference | 331 | discriminative | 1611 | exclude | 764 |
| discriminate | 856 | excluding | 268 | biases | 1535 | excluding | 601 |

HMS – Health and Medical Sciences, LS – Life Sciences, PS – Physical sciences, SS&E – Social Sciences and Economics

**Table 26.** Ten most frequent words found in review reports for Loyalty-Virtue across journal’s fields of research

| **Loyalty-Virtue (HMS, total=105)** | | **Loyalty-Virtue (LS, total=53)** | | **Loyalty-Virtue (PS, total=120)** | | **Loyalty-Virtue (SS&E, total=110)** | |
| --- | --- | --- | --- | --- | --- | --- | --- |
| **Word** | **Freq.** | **Word** | **Freq** | **Word** | **Freq** | **Word** | **Freq** |
| group | 98862 | group | 15979 | group | 37110 | group | 14718 |
| cohort | 19703 | together | 1992 | community | 28047 | family | 9371 |
| joint | 13174 | family | 1351 | together | 19775 | community | 7770 |
| community | 9409 | community | 851 | communities | 10298 | national | 3954 |
| together | 7082 | cohort | 575 | national | 5737 | together | 3542 |
| family | 6389 | families | 383 | family | 4852 | communities | 2677 |
| national | 5909 | national | 216 | devoted | 4734 | families | 2197 |
| families | 2555 | joint | 213 | joint | 4151 | cohort | 2002 |
| united | 2233 | segregation | 203 | united | 1979 | loyalty | 1524 |
| communities | 1397 | member | 190 | families | 1840 | collective | 1141 |

HMS – Health and Medical Sciences, LS – Life Sciences, PS – Physical sciences, SS&E – Social Sciences and Economics

**Table 27.** Ten most frequent words found in review reports for Loyalty-Vice across journal’s fields of research

| **Loyalty-Vice (HMS, total=64)** | | **Loyalty-Vice (LS, total=34)** | | **Loyalty-Vice (PS, total=68)** | | **Loyalty-Vice (SS&E, total=69)** | |
| --- | --- | --- | --- | --- | --- | --- | --- |
| **Word** | **Freq.** | **Word** | **Freq** | **Word** | **Freq** | **Word** | **Freq** |
| individual | 14326 | individual | 2955 | individual | 22071 | individual | 12594 |
| individuals | 14222 | individuals | 2671 | individuals | 9873 | individuals | 9179 |
| individually | 927 | individually | 222 | individually | 2018 | immigrants | 1956 |
| foreign | 667 | foreign | 152 | abandoned | 1217 | foreign | 1603 |
| individualized | 354 | immigration | 45 | abandonment | 606 | immigrant | 1513 |
| immigrants | 278 | immigrants | 40 | foreign | 550 | immigration | 663 |
| immigrant | 253 | immigrant | 25 | enemies | 482 | individually | 347 |
| abandoned | 206 | abandoned | 22 | immigration | 419 | individualism | 287 |
| immigration | 148 | abandon | 15 | abandon | 174 | individualistic | 198 |
| enema | 115 | individualized | 14 | sequester | 155 | betrayal | 189 |

HMS – Health and Medical Sciences, LS – Life Sciences, PS – Physical sciences, SS&E – Social Sciences and Economics

**Table 28.** Ten most frequent words found in review reports for Authority-Virtue across journal’s fields of research

| **Authority-Virtue (HMS, total=241)** | | **Authority-Virtue (LS, total=136)** | | **Authority-Virtue (PS, total=390)** | | **Authority-Virtue (SS&E, total=187)** | |
| --- | --- | --- | --- | --- | --- | --- | --- |
| **Word** | **Freq.** | **Word** | **Freq** | **Word** | **Freq** | **Word** | **Freq** |
| control | 46573 | control | 12342 | order | 87781 | control | 12109 |
| order | 20365 | order | 5671 | control | 64026 | status | 10374 |
| status | 17609 | respect | 2037 | respect | 27419 | order | 10156 |
| respect | 8731 | position | 1785 | submitted | 20344 | respect | 3491 |
| position | 8435 | status | 1406 | class | 18830 | position | 2973 |
| submitted | 5773 | submitted | 1348 | position | 15560 | class | 2657 |
| submission | 5680 | submission | 882 | submission | 14994 | mothers | 2657 |
| class | 3874 | class | 818 | traditional | 10472 | traditional | 2046 |
| compliance | 3670 | serve | 390 | status | 9592 | loyalty | 1524 |
| traditional | 3643 | traditional | 358 | submit | 5024 | leadership | 1497 |

HMS – Health and Medical Sciences, LS – Life Sciences, PS – Physical sciences, SS&E – Social Sciences and Economics

**Table 29.** Ten most frequent words found in review reports for Authority-Vice across journal’s fields of research

| **Authority-Vice (HMS, total=56)** | | **Authority-Vice (LS, total=32)** | | **Authority-Vice (PS, total=70)** | | **Authority-Vice (SS&E, total=82)** | |
| --- | --- | --- | --- | --- | --- | --- | --- |
| **Word** | **Freq.** | **Word** | **Freq** | **Word** | **Freq** | **Word** | **Freq** |
| refuse | 220 | illegal | 18 | illegal | 817 | illegal | 366 |
| agitation | 144 | refuse | 18 | agitation | 782 | betrayal | 189 |
| illegal | 136 | agitation | 17 | refuse | 262 | refuse | 133 |
| oppose | 110 | oppose | 14 | oppose | 185 | protest | 74 |
| agitated | 70 | subvermispora | 5 | agitated | 141 | oppose | 69 |
| obstruct | 24 | defy | 4 | illegally | 84 | rebel | 30 |
| defy | 19 | agitated | 3 | obstruct | 59 | illegally | 28 |
| illegally | 18 | disrespectful | 3 | agitator | 40 | disrespectful | 26 |
| dissent | 15 | obstruct | 3 | protest | 37 | denounce | 24 |
| disrespectful | 14 | rebelato | 3 | defy | 30 | disrespect | 24 |

HMS – Health and Medical Sciences, LS – Life Sciences, PS – Physical sciences, SS&E – Social Sciences and Economics

**Table 30.** Ten most frequent words found in review reports for Sanctity-Virtue across journal’s fields of research

| **Sanctity-Virtue (HMS, total=107)** | | **Sanctity-Virtue (LS, total=58)** | | **Sanctity-Virtue (PS, total=188)** | | **Sanctity-Virtue (SS&E, total=93)** | |
| --- | --- | --- | --- | --- | --- | --- | --- |
| **Word** | **Freq.** | **Word** | **Freq** | **Word** | **Freq** | **Word** | **Freq** |
| purely | 1502 | pure | 585 | pure | 13154 | pure | 585 |
| pure | 1288 | integrity | 556 | purity | 5966 | clean | 570 |
| integrity | 1000 | purely | 351 | clean | 5930 | purely | 569 |
| clean | 756 | upright | 135 | cleaning | 4324 | austerity | 298 |
| refined | 573 | clean | 125 | purely | 2831 | refined | 268 |
| upright | 474 | purity | 121 | integrity | 2236 | church | 216 |
| abstinence | 403 | sterile | 84 | refined | 2230 | decentralization | 209 |
| preserve | 361 | refined | 74 | cleaned | 1521 | integrity | 176 |
| purity | 351 | preserve | 52 | pristine | 1363 | cleaner | 165 |
| sterile | 295 | decent | 46 | cleanup | 1187 | decent | 160 |

HMS – Health and Medical Sciences, LS – Life Sciences, PS – Physical sciences, SS&E – Social Sciences and Economics

**Table 31.** Ten most frequent words found in review reports for Sanctity-Vice across journal’s fields of research

| **Sanctity-Vice (HMS, total=117)** | | **Sanctity-Vice (LS, total=53)** | | **Sanctity-Vice (PS, total=158)** | | **Sanctity-Vice (SS&E, total=98)** | |
| --- | --- | --- | --- | --- | --- | --- | --- |
| **Word** | **Freq.** | **Word** | **Freq** | **Word** | **Freq** | **Word** | **Freq** |
| disease | 37275 | disease | 5273 | disease | 9897 | disease | 3556 |
| diseases | 6342 | diseases | 1397 | stainless | 4290 | diseases | 1268 |
| staining | 4760 | staining | 1361 | diseases | 3448 | sick | 655 |
| gross | 1008 | stained | 245 | exploitation | 1813 | exploitation | 599 |
| stain | 877 | stain | 215 | exploited | 1719 | sickness | 599 |
| sick | 849 | gross | 147 | staining | 1238 | exploit | 376 |
| stained | 810 | stains | 106 | exploit | 1164 | contagion | 345 |
| sicker | 685 | diseased | 88 | gross | 1162 | gross | 224 |
| diseased | 678 | exploited | 77 | exploiting | 932 | exploitative | 182 |
| sickle | 570 | disgust | 64 | repulsion | 711 | exploited | 176 |

HMS – Health and Medical Sciences, LS – Life Sciences, PS – Physical sciences, SS&E – Social Sciences and Economics
